# Glyco-binding domain chimeric antigen receptors as a new option for cancer immunotherapy

**DOI:** 10.1038/s41434-022-00374-x

**Published:** 2022-12-19

**Authors:** Anna-Katharina Franke, Charlotte Wessolowski, Vanessa Thaden, Ingo Müller, Kerstin Cornils

**Affiliations:** 1 Research Institute Children’s Cancer Center Hamburg, Hamburg, Germany; 2 Department of Pediatric Hematology and Oncology, Division of Pediatric Stem Cell Transplantation and Immunology, University Medical Center Hamburg-Eppendorf, Hamburg, Germany

**Keywords:** Immunotherapy, Breast cancer

## Abstract

In the last decade, treatment using Chimeric Antigen Receptor (CAR) are largely studied and demonstrate the potential of immunotherapeutic strategies, as seen mainly for blood related cancers. Still, efficient CAR-T cell approaches especially for the treatment of solid tumors are needed. Tn- and Sialyl-Tn antigens are tumor associated carbohydrate antigens correlating with poor prognosis and tumor metastasis on a variety of tumor entities. These glycans can be recognized by CD301 (CLEC10A, MGL), which is a surface receptor found primarily on immune cells. In the present study, we hypothesized, that it is possible to use newly generated CD301-bearing CARs, enabling cytotoxic effector cells to recognize and eliminate breast cancer cells. Thus, we genetically modified human NK92 cells with different chimeric receptors based on the carbohydrate recognition domain (CRD) of human CD301. We assessed their cytotoxic activity in vitro demonstrating the specific recognition of CD301 ligand positive cell lines. These results were confirmed by degranulation assays and in cytokine release assays. Overall, this study demonstrates CD301-CARs represent a cost-effective and fast alternative to conventional scFv CARs for cancer immunotherapy.

## Introduction

One of the most characteristic features of cancer cells is altered glycosylation capacities that result in exposure of aberrant glycans on the surface of the cells [1]. One prominent example is the Tn-antigen, which is defined as a N-acetylgalactosamine (GalNAc) residue bound to serine or threonine by an α-glycosidic linkage [2] representing the initial step of O-linked glycan synthesis [3, 4]. The Tn-structure may further serve as an acceptor for the sialyltransferase ST6GalNAc-I, resulting in sialyl-Tn (STn).The Tn and/or STn structures are found to be overexpressed on glycoproteins and mucins on the surface of a variety of solid tumors e.g. prostate cancer, ovarian carcinoma, endometrial cancer, colorectal cancer or breast cancer [5]. It has been suggested that the expression of Tn and/or STn structures on tumor cells are accompanied by an increased rate of local recurrences and distant metastases [6]. However, frequencies of Tn − /STn- detection and correlation with patients’ outcome vary considerably between studies, which may be explained by the variable specificities of antibodies and lectins used for detection and the compositions of the patient cohorts [7, 8].

Tumor-associated antigens on the surface of cancer cells have been used as targets for immunotherapy by the utilization of T cells expressing chimeric antigen receptors (CAR). These molecules target the epitopes by a single-chain antibody-fragment (scFv) derived from monoclonal antibodies and lead to a killing of the tumor cells by the perforin/granzyme pathway and the death receptor pathway [9]. Based on the clinical success of CD19-based CAR T cell therapy, a variety of different CARs have been generated and tested for different cancer entities [10]. The altered glycan-pattern on tumor cells has also come into focus of CAR-T cell based immunotherapy. In the nineties, a first generation CAR specific for tumor-associated glycoprotein 72 (TAG72), which is a STn O-glycan hapten, was first reported to efficiently target gastrointestinal tumor cell lines [11, 12]. However, in patients with metastatic colorectal cancer receiving CAR-T cell therapy clinical responses were not observed, presumably due to rejection of the CAR-T cells caused by CAR antigenicity or the lack of co-stimulatory domains [13].

After further developments, a second-generation antibody-based CAR, using the antibody 5E5 targeting the Tn-MUC1 epitope, specifically eliminated pancreatic- and leukemia cells in xenograft models. The CAR displayed cancer-specificity and negligible reactivity against normal tissues and is actually tested in a phase I human clinical trial [14, 15].

The applications of these antibody-based CARs are limited to cancers expressing a specific glycan (anti-TAG72 CAR) or to tumors presenting glycans on a specific protein like Mucin 1 (5E5). The use of human lectin domains for target recognition would circumvent these problems, since lectins recognize several close related glycan structures independently of the carrier protein. Such an approach was published by Meril et al., who developed CARs incorporating the exodomains of human Siglec-7 and Siglec-9 to bind sialoglycans [16]. Siglec-based CAR T-cells were able to mediate antitumor activity against cell lines derived from leukemia and ovarian cancer histotypes in vitro as well as a patient-derived melanoma xenograft mouse model.

Another family of human lectins encompasses the C-type lectins, having a diverse range of functions including cell-cell adhesion and immune response regulation. They harbor a carbohydrate recognition domain (CRD) with distinct specificity for the glycan epitopes [17]. We hypothesized that the utilization of the CRD might serve as an alternative strategy for targeting these glycans in human tumors overcoming some of the limitations postulated for scFv-based CARs. In the presented study here, we developed a newly CAR construct by utilization of the CRD of the physiologically expressed glycoreceptor CD301 as the targeting domain. CD301 (MGL, CLEC10A) is a member of C-type lectin family expressed by dendritic cells (DCs) and macrophages. It preferentially binds terminal GalNAc structures such as the Tn- and STn-antigens and is involved in immune response modulation [18–23]. We have already shown that CD301 is able to detect ligands relevant for cancer prognosis in breast cancer tissue sections [8, 24]. Our data presented here, show the functionality of our newly developed CD301-CAR on Tn/STn-positive breast cancer cell lines.

## Material & methods

Detailed information about the experimental procedure for cultivation of cell lines, FACS Analysis of cell lines, glycan binding assay, degranulation assay and interferon gamma (IFN-γ) ELISA are given in the supplement. All experiments were performed at least two times. Independent biological repeats for degranulation assay and IFN-γ secretion are also shown in the supplement.

### Generation of CAR-expressing NK92 cells

**Fig. 1:**
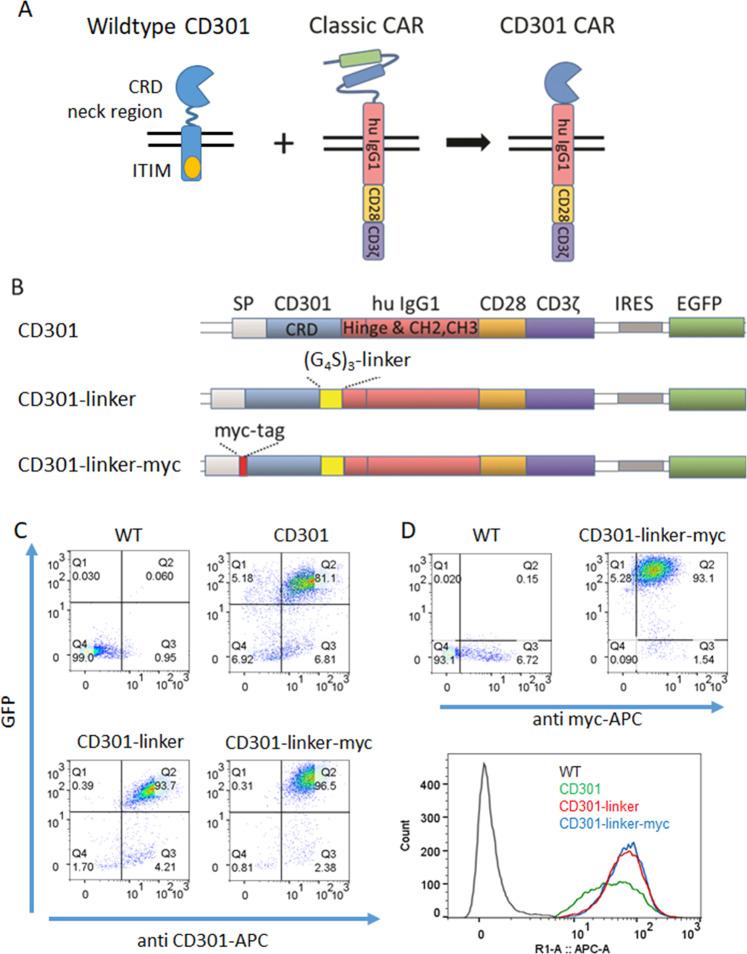
CD301-based CAR construction and expression in NK92 cells. **A** To generate the CD301 CAR, we used the Moloney murine leukemia virus-derived retroviral pBullet expression vector [25] and exchanged the scFv-sequences with the CRD of CD301. The newly generated fusion protein contained the kappa leader sequence as a signal peptide (SP), the CD301-CRD, the human Fc-part of an IgG (hinge and hu IgG) the transmembrane and intracellular domain of human CD28 and the signaling domain of human CD3ζ. We additionally quipped the vector with a GFP linked by an IRES. **B** Schematic representation of the different CD301 based CARs: we generated the CD301 linker by addition of a [(G_4_S)_3_] linker downstream of the CRD to increase flexibility. **C** NK92 cells were retrovirally transduced and sorted based on the GFP expression. The expression of the CD301 CAR construct was measured by flow cytometry using antibody specific for the myc-tag and the CD301 CRD, respectively. **D** FACS analysis of transduced NK92 cells using anti-myc antibody. **E** Overlay histogram of CD301 CAR expressions.

The scFv-domain of a retroviral second-generation CAR-construct [25] was exchanged by the CRD of CD301 and additionally linked to an eGFP via an internal ribosomal entry site (IRES) (Fig. 1A). To generate the different CD301-CAR-constructs, we inserted a glycine-serine linker [(G_4_S)_3_] and a myc-tag (Fig. 1B). For virus production, the retroviral expression vector DNA was cotransfected with the retroviral helper plasmids DNAs phCMV-GALV and pcDNA3.1MLV.gp into HEK293T cells using Lipofectamine 3000 (Thermo Fisher Scientific). Supernatants were directly used to transduce human NK cell line NK92 (Passage number 7). Transduction efficiency was determined by eGFP-expression and/or additional staining with anti-CD301 (CLEC10A) antibody (Biolegend, # 354705) or anti-myc-antibody (Santa Cruz, sc-40 AF647) via flow cytometry. Seven days after transduction, cells were sorted and the subsequent experiments were performed until passage number 30.

### Cytotoxicity measurements

The cytotoxic activity of NK92 CAR and parental NK92 cells was analyzed in a standard 3 h calcein release assay [26]. In brief, effector cells were incubated with 1 × 10^4^ calcein-labeled target cells at various E:T ratios in quadruplicate wells of 96-well round-bottom plates. The percentage of specific calcein release into the supernatant was calculated from spontaneous lysis in wells with target cells only and maximal lysis obtained in wells containing 1% TritonX-100.

### Statistics

Data were assessed for similarity of variance and statistical analysis was performed by unpaired two-tailed Student’s t-test. Horizontal bars represent means ± standard deviation. *p* < 0.05, *p* < 0.01 or *p* < 0.001 p < 0.0001 were considered statistically significant and indicated by *, **, *** or **** respectively.

## Results

### Generation and expression of CD301-CAR constructs

In order to generate a CAR with the same glycan-specificity we exchanged the scFV of a second generation CAR [25] with the CRD of CD301. We additionally equipped the γ-retroviral vector with an eGFP as reporter. In a similar construct, we cloned a CD301-CAR construct with a threefold Glycine-Serine-linker [(G_4_S)_3_] between the CRD and the IgG to increase the flexibility of the domain (CD301-linker). To investigate the impact of a N-terminal myc-tag in binding properties of a CRD-containing CAR we also fused a myc-tag at the 5ʹ-end of the CD301 CRD (CD301-linker-myc) (Fig. 1A + B). All viral constructs were expressed in NK92 cells to evaluate the expression and correct orientation of the CD301-CAR construct. The transduction efficiencies varied from 2.2% to 6.4%. After sorting, we performed flow cytometric analysis using an anti CD301 antibody specific for the CRD region to recognize the CAR constructs (Fig. 1C + E). All three CAR constructs were detected at the cell surface with comparable expression levels ranging from 81 to 96%, respectively. The use of an anti-myc antibody revealed a comparable expression level (96%) of the CD301-linker-myc construct as we observed with the anti-CD301 antibody (Fig. 1D). All constructs showed also stable expression after extended period of cultivation (Supplementary Fig. 1A + B)

### Binding capacity of CD301-CAR constructs

**Fig. 2:**
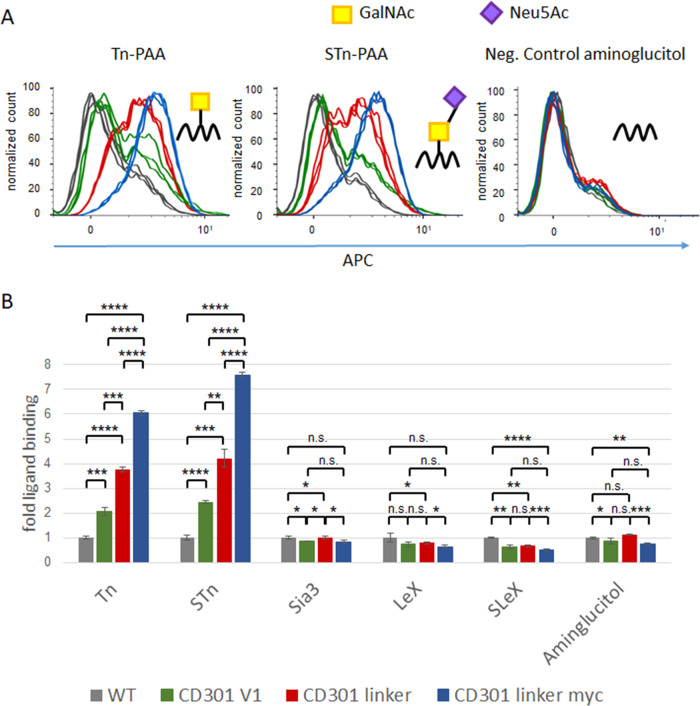
Binding properties of CD301 CARs. **A** Binding assay with fluorescently labeled PAA-glykoconjugates. NK92 cells expressing the respective CARs were incubated with biotinylated PAA-glycoconjugates premixed with Streptavidin-Alexa 647 and analyzed by flow cytometry. Binding of Tn- PAA, STn-PAA or the negative control (aminoglucitol) to NK92 cells are depicted as triplicates (WT: grey, CD301 CAR: green, CD301-linker: red, CD301-linker-myc: blue). **B** Evaluation of binding specificity. Binding of indicated PAA-glycoconjugates are shown as mean of fold binding compared to WT cells. Error bars showing standard deviation of triplicates. *p* < 0.05, *p* < 0.01 or *p* < 0.001 p < 0.0001 were indicated by *, **, *** or **** respectively.

After determination of the expression of the CD301-CAR constructs, we tested the binding properties and specificity of the CRD domain fused to the CAR backbone. To do so, we made use of fluorescently labeled glycoconjugates (Tn and STn) fused to polyacrylamide (PAA) in a FACS-based binding assay (Fig. 2A). Compared to the negative controls aminoglucitol and untransduced NK92 cells (grey histograms), we observed significant binding of Tn-PAA and STn-PAA to all CD301-CARs. In addition, no binding of other glycan-structures, like Sia3-, Lewis X (Le^x^)- or Sialyl-Le^x^ were detected (Fig. 2B). CD301-linker-CARs (red and blue histograms) showed an enhanced binding of Tn- and STn-PAA compared to CD301-CAR without linker (green histograms) resulting in an increase of binding from two to six fold compared to wildtype cells. STn binding was enhanced in a similar range (CD301-CAR: 2-fold, CD301-linker: 4-fold, CD301-linker-myc: 7-fold). Taken together, our data showed that the CRD of CD301 specifically binds to its glycan-structure ligands.

### NK92 cells expressing CD301-CARs can mediate antitumor activity against several targets

**Fig. 3:**
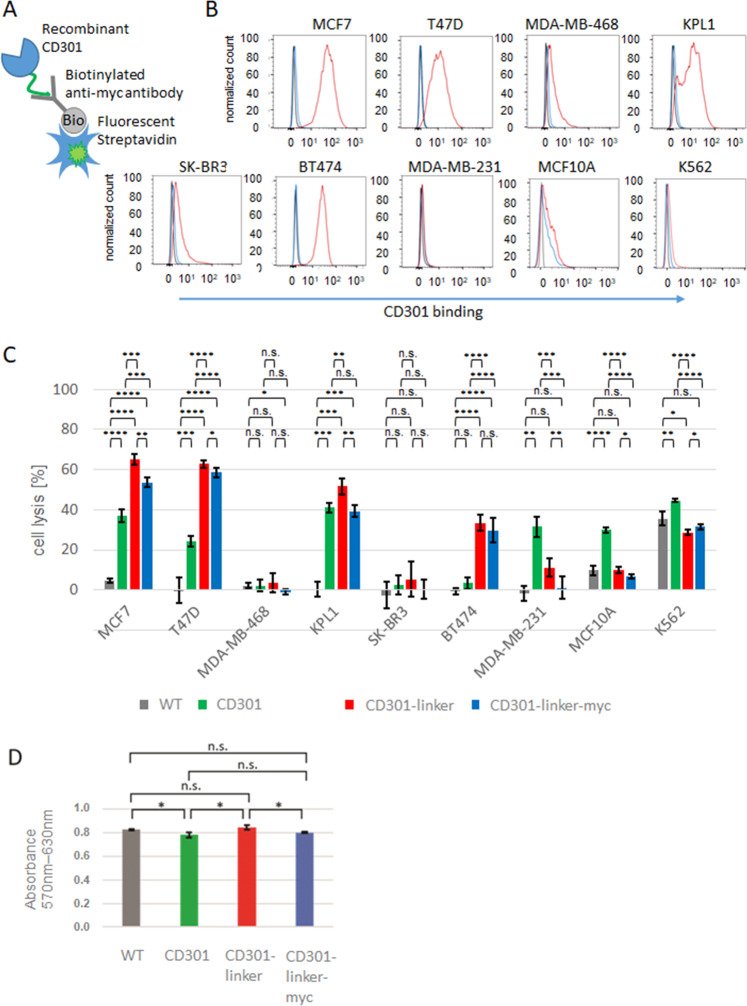
CD301-CARs display specific cytotoxicity against breast cancer cell lines. **A** Recombinant CD301 is equipped with a N-terminal myc and was fluorescently labeled with a biotinylated anti-myc antibody and Strepatividin-FITC. **B** Detection of CD301 ligands on target cells: breast cancer cell lines were stained with fluorescently labeled recombinant CD301 and analyzed by flow cytometry. **C** NK92 cells expressing the CD301-CAR, CD301-linker-CAR or CD301-linker-myc CAR were cocultured for 3 h with calcein labeled targets with an effector target ratio of 5:1 and measured as quadruplicates. **D** MTT assay of transduced - and WT NK92 cell. Columns represent the median of triplicates. Error bars show standard deviation. *p* < 0.05, *p* < 0.01 or *p* < 0.001 p < 0.0001 were indicated by *, **, *** or **** respectively.

Next, we wanted to test, if our newly generated fusion constructs are able to transmit the signals for degranulation and killing after binding to target cells. In order to prove this, seven different breast cancer cell lines and one human mammary epithelial cell line (MCF10A) were screened for the expression of CD301 ligands at the cell surface by staining with a recombinant, soluble CD301 and analyzed in flow cytometry (Fig. 3A, B). High expression of Tn or STn antigens were detected on estrogen receptor positive cell lines MCF7, T47D, KPL1 and BT474, whereas the estrogen receptor negative cell lines MDA-MB-468 and MDA-MB-231(triple negative), SK-BR3 (Her2 positive),the non-tumorigenic epithelial cell line MCF10A and the myelogenous leukemia cell line K562 showed lower expression or were negative (Fig. 3B).

Consequently, we evaluated the lytic activity of the NK92/CAR cells towards the different breast cancer cell lines with an E:T ratio of 5:1 (Fig. 3C). Compared to untransduced counterparts, NK92 cells expressing CD301-CARs showed increased cytotoxicity towards Tn/STn positive cell lines MCF7, T47D, KPL1 and BT474, whereas the negative cell lines or cells with low ligand expression were less effectively killed. Exceptions are the cell lines MDA-MB-231 and MCF10A, to which the CD301 construct shows a significant cytotoxicity. In a direct comparison, we see more specific cytotoxic activity of the constructs with linker sequences (and myc-tag) and improved correlation of killing efficiency and ligand expression (Supplementary Fig. 3). As NK92 cells exhibit killing activity against K562 cell line lacking the MHC complex required to inhibit NK activity, we compared the natural cytotoxicity of the wildtype and the transduced populations. NK92 WT and NK92 CAR cells disclosed a relevant lysis of K562 cells indicating that the transduction and selection procedures do not affect the cytotoxic properties of NK92 cells.

Parallel to cytoxicity measurements we assessed the cell metabolic activity as a marker for vitality of the effector cells in MTT assay. We detected no differences between the different NK92 cell lines (Fig. 3D). Thus, the results obtained from cytotoxicity assays indicated a specific killing of the CD301-CARs constructs.

**Fig. 4:**
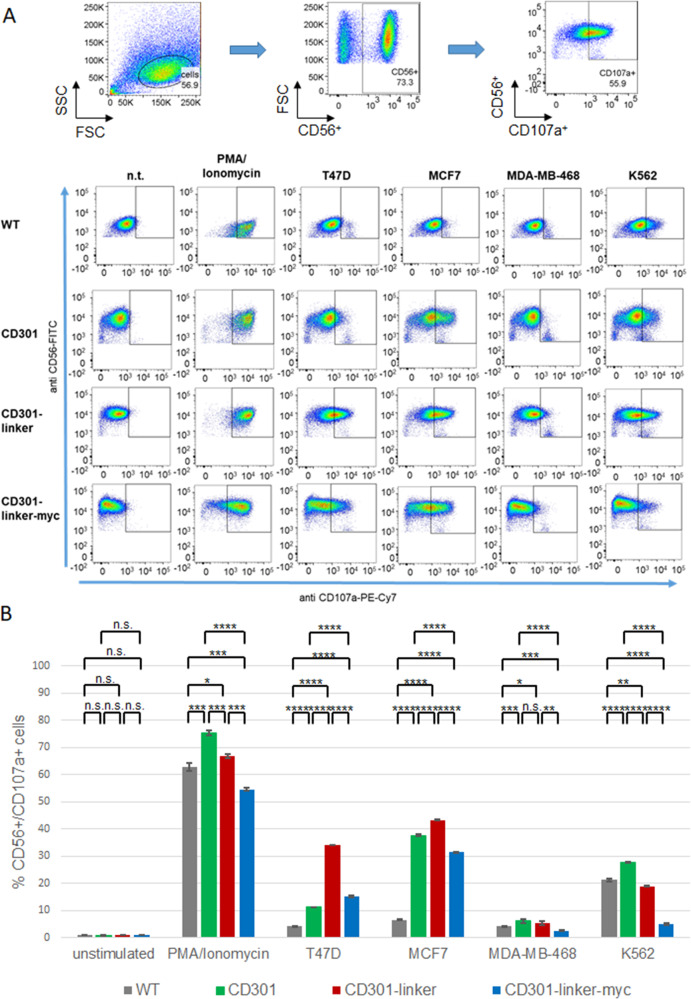
CAR expression leads to enhanced degranulation of NK92 cells upon engagement with CD301 ligand-positive breast cancer cells. **A** Degranulation of CD56^+^ NK92 CAR cells was analyzed by flow cytometry assessment of CD107a surface expression after 4 h of co-culture with MCF7, T47D, MDA-MB-468 or K562 cells (CD56^-^) (E:T 1:1). Parental NK92 cells were included for comparison. Unstimulated effector cells or stimulated with PMA/ionomycin served as basal and positive controls, respectively. **B** Evaluation of degranulation assay. Columns represent mean percentage of CD107a positive NK92 cells measured in triplicates. Error bars show standard deviation. *p* < 0.05, *p* < 0.01 or *p* < 0.001 p < 0.0001 were indicated by *, **, *** or **** respectively.

The secretion of lytic granules results in surface exposure of the lysosomal-associated protein CD107a. As shown in Fig. 4, we analyzed the percentage of CD107a positive NK92 cells after incubation with different target cell lines.

By using Tn/STn -expressing breast cancer cell lines T47D and MCF7, NK92 CAR cells underwent a strong upregulation of CD107a expression that was very limited in wildtype NK92 cells. Although the percentage of GFP-positive NK92 cells were comparable (Supplementary Fig. 5A), the degranulation levels in the presence of T47D cells varied between the CAR constructs. Linker CARs led to a more effective degranulation compared to CD301-CAR without linker, presumably due to linker-dependent improved accessibility of the target structures.

Importantly, the incubation with ligand-negative MDA-MB-468 cells only led to a slight increase of CD107a positive cells demonstrating the specificity of the newly generated CAR constructs.

PMA/ionomycin treatment was used as a control for maximal degranulation. For measuring CAR-independent degranulation, NK92 cells were incubated with K562 cells. In contrast to the other NK92 cell lines this non-specific stimulus produced only marginal effects in NK92 cells expressing CD301-linker-myc CAR, although these cells still show enhanced degranulation towards MCF7 and T47D cells.

A common feature of antigen receptors like T cell receptor and synthetic CARs is ligand-induced downmodulation considering a hallmark of lymphocyte activation [27]. Therefore, we investigated surface expression of CD301-CARs after incubation with target cells for 3- or 24 h, respectively, PMA treatment was included as a control for activation. To investigate the impact of degradation by proteases (sheddases), we applied in parallel TNF protease inhibitor 2 (TAPI-2), a broad-spectrum inhibitor of sheddases.TAPI-2 inhibits PMA-induced shedding of various cell surface proteins [28–30].

We observed downregulation of 40–65% of surface CD301-CARs on NK92 cells incubated with CD301 ligand positive target cells after 3 h. The strongest effect was measureable after incubation of linker CD301-CAR expressing NK92 cells with T47D, which was not as pronounced in NK92 cells expressing CD301-CAR without linker sequence. MDA-MB-468 cells providing only low levels of CD301 ligands and induced only slight downregulation of the linker CD301-CARs. Compared to NK92 cells treated with PMA alone, the addition of TAPI-2 led to an increase of ~5%. After 24 h, a partial recovery of the surface CD301-linker-CARs was detectable, while the amount of CD301-CARS without linker remained reduced. To reveal, if CD301 domains are cleaved from the CAR constructs, we performed competing binding assays using the supernatants after 24 h co-culture in combination with fluorescently labeled recombinant CD301 (Supplementary Fig. 4). We measured only slight inhibitory effects of maximal 10% reduction of binding to MCF7 cells. These results lead us to the assumption, that the major part of down-regulation is not due to degradation by sheddases.

### CD301-CARs are selectively activated by target structures on cancer cells resulting in a different extent of activation

To further confirm that the increased killing of NK92 CAR cells was strictly Tn/STn-specific and could be ascribed only to CAR activation, we analyzed cytotoxicity using different E:T ratios. NK92 CD301-CAR cells showed, even at low E:T ratios, an extremely high cytotoxicity to Tn/STn-positive MCF7 and T47D cells, which were not killed by wildtype NK92 cells. The differences between the CAR constructs were only marginal within a range of 20%. CD301-linker-CAR turned out to be the most effective construct (Fig. 6A). These tendencies was also observed after long term cultivation of NK92 cells indicating robust performance of the NK92 CAR cells (Supplementary Fig. 1C).

**Fig. 5:**
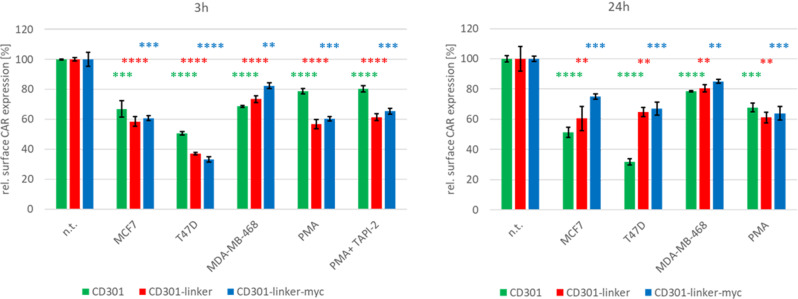
Ligand induced downregulation of CAR constructs. Surface expression of CD301 CARs were analysed after 3 h or after 24 h of co-culture with MCF7, T47D, MDA-MB-46. PMA treated cells served as postiv control for activation. In parallel 50 µM TAPI-2, an inhibitor for MMPs and TACE, was added to PMA treated cells for 3 h. Error bars show standard deviation. Significance levels were calculated in relation to non-treated cells (n.t.) *p* < 0.05, *p* < 0.01 or *p* < 0.001 p < 0.0001 were indicated by *, **, *** or **** respectively.

Likewise, T47D cells were also selectively killed by NK92 CAR cells but not by the wildtype NK92 counterparts (Fig. 5A). Again, CD301-linker-CAR showed the highest lytic activity. In contrast NK92 expressing CD301-CAR or CD301-linker-myc-CAR mediated significantly lower killing of T47D cells. As further proof of specificity, both NK92 CAR and NK92 WT cells failed to lyse efficiently Tn/STn-negative MDA-MB-468, MCF10A cells and three different neuroblastoma cell lines (Supplementary Fig. 6).

**Fig. 6:**
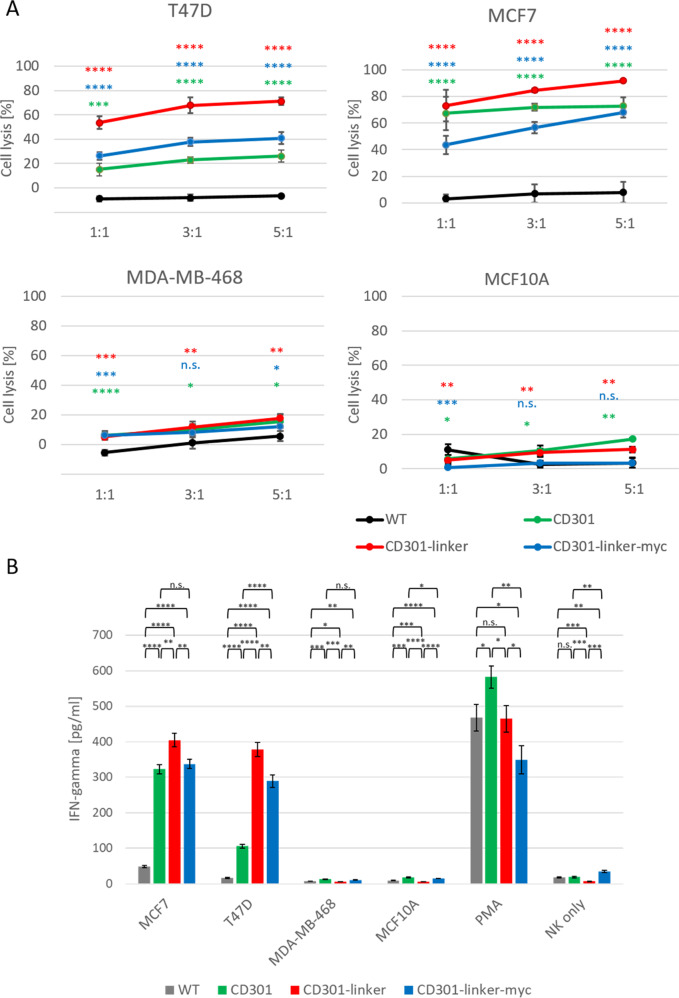
Lytic activity of CD301-CAR expressing NK92 cells. **A** Cytotoxicity of NK92 cell expressing CD301-CAR (green), CD301-linker-CAR (red) or CD301-linker-myc CAR (blue) in comparison to wildtype NK92 cells (grey) was investigated against MCF7, T47D, MDA-MB-468 cells and MCF10A, at different E:T ratios. **B** IFN-γ release was analyzed in supernatants of CAR expressing NK92 CAR or wildtype NK92 cells stimulated with MCF7, T47D, MDA-MB-468 cells and MCF10A (E:T 1:1), respectively. Untreated effector cells and effector cells treated with PMA/ionomycin served as controls. Results are reported as mean values ± SD of triplicates. *p* < 0.05, *p* < 0.01 or *p* < 0.001 p < 0.0001 were indicated by *, **, *** or **** respectively.

Additionally, we tested IFN-γ release by NK92 CAR and NK92 cells following target engagement. Results of this assay fully mirrored those already obtained with CD107a expression analysis shown in Fig. 4, as PMA/ionomycin massively, but non-specifically, stimulated either effector populations. MDA-MB-468 and MCF10A cells were negligibly recognized, while CAR expressing NK92 cells produced significant levels of IFN-γ upon interaction with CD301 ligand positive cell lines MCF7 and T47D (Fig. 6B). Again, CD301-linker-CAR induces the highest IFN-γ secretion especially in the presence of T47D.

The data indicates that the lectin based CD301-CAR is fully functional and confers glycan-selective directed and enhanced activity.

## Discussion

Immunotherapy can provide an indisputable clinical benefit in advanced cancer patients. The present study is the first investigating the use of a C-type lectin based CAR for targeting a distinct glycosylation pattern associated with cancer. Based on previous studies [8, 24], we used CD301 and designed a panel of CD301-CRD based CAR constructs to detect cancer‐related glycans predominantly, Tn- and STn-Antigens. Marcelo et al. reported varying binding properties of short – and long CD301 isoforms [31]. We therefore generated two different versions of the CD301-CAR construct: one with a direct fusion to IgG, the other with a flexible G_4_S-linker to target other Tn/STn, which might be less accessible. Indeed our results suggest the presentation of target structures with distinct accessibilities on the surface of MCF7 and T47D cells. The insertion of the linker significantly improved glycan binding and cytotoxicity of the CARs, especially to T47D cells even at low E:T ratios.

The full functionality of this CAR confers ligand-specific and increased lytic activity but also leads to significant IFN-γ secretion, which would stimulate in vivo the recruitment of other immune cells to enhance the antitumor response.

We have shown previously that CD301 ligands can be further induced in hormone-dependent cells like MCF7 and T47D cells by tamoxifen treatment [8]. Thus, it may be important to combine CD301‐based CARs with other treatments to fully achieve higher antitumor function.

In our hands we observed killing of ligand negative cells like MDA-MB-231, MDA-MB-468 and MCF10A cells especially with the CD301 CAR without linker. The insertion of the linker sequence improved the specificity of the CARs, but further analysis have to address toxic effects towards ligand positive and negative noncancerous tissues. Since CD301 ligands can be also detected in normal tissue like the gastrointestinal tract, it may be crucial to combine CD301‐CAR with other approaches to modulate CAR activity. The coexpression with an inhibitory CAR [32] could minimize off-target effects. Alternatively, the use of “AND gates” or the inclusion of suicide genes could also increase the safety of this CAR [33].

On the other hand, the TN-MUC1 antibody 5E5 used for CAR generation by Posey et al. exhibited a similar binding pattern on human tissue array like recombinant CD301. The corresponding CAR is actually tested in a human Phase I study to evaluate the safety and preliminary efficacy of CART-TnMUC1-Cells for the treatment of solid-tumors [8, 14, 15]. Although the study is still early in dose-escalation phase, having completed only 2 of 6 planned dose levels, no evidence of safety concerns or severe on-target/off-tumor toxicity was observed so far.

Taken together, the lectin-based CD301-CARs could represent an alternative strategy to antibody-based CARs for targeting cancer associated glycan-structures. The use of human receptor domains could reduce immunogenicity of CARs observed in some studies due to recognition of the murine-based scFvs by the immune system [34]. Another advantage of this variant of CARs is, that they are faster to generate than scFv-CARs, because the time-consuming process of antibody generation does not take place. Moreover the application is not limited to a certain cancer type, because lectin binding is not limited to a specific glycopeptide and the addressed glycans Tn- and STn-antigen are expressed in multiple carcinomas making the CAR applicable to the appropriate cancer entities.

## Supplementary information

Supplemental material and methods

## Data Availability

The data that support the findings of this study are available on request from the corresponding author.

## References

[1] Hakomori S (2002). Glycosylation defining cancer malignancy: new wine in an old bottle. Proc Natl Acad Sci USA.

[2] Dahr W, Uhlenbruck G, Gunson HH, van der Hart M (1975). Studies on glycoproteins and glycopeptides from Tn-polyagglutinable erythrocytes. Vox Sang.

[3] Brockhausen I, Schachter H, Stanley P O-GalNAc Glycans. In: nd, Varki A, Cummings RD, Esko JD, Freeze HH, Stanley P, et al., editors. Essentials of Glycobiology. Cold Spring Harbor (NY) 2009. 20301232

[4] Ju T, Otto VI, Cummings RD (2011). The Tn antigen-structural simplicity and biological complexity. Angew Chem Int Ed Engl.

[5] Fu C, Zhao H, Wang Y, Cai H, Xiao Y, Zeng Y (2016). Tumor-associated antigens: Tn antigen, sTn antigen, and T antigen. HLA..

[6] Ju T, Wang Y, Aryal RP, Lehoux SD, Ding X, Kudelka MR (2013). Tn and sialyl-Tn antigens, aberrant O-glycomics as human disease markers. Proteomics Clin Appl.

[7] Julien S, Videira PA, Delannoy P (2012). Sialyl-tn in cancer: (how) did we miss the target?. Biomolecules..

[8] Kurze AK, Buhs S, Eggert D, Oliveira-Ferrer L, Muller V, Niendorf A (2019). Immature O-glycans recognized by the macrophage glycoreceptor CLEC10A (MGL) are induced by 4-hydroxy-tamoxifen, oxidative stress and DNA-damage in breast cancer cells. Cell Commun Signal.

[9] Lemoine J, Ruella M, Houot R (2021). Overcoming intrinsic resistance of cancer cells to CAR T-cell killing. Clin Cancer Res.

[10] June CH, O’Connor RS, Kawalekar OU, Ghassemi S, Milone MC (2018). CAR T cell immunotherapy for human cancer. Science.

[11] Hombach A, Heuser C, Sircar R, Tillmann T, Diehl V, Kruis W (1997). T cell targeting of TAG72+ tumor cells by a chimeric receptor with antibody-like specificity for a carbohydrate epitope. Gastroenterology..

[12] Hombach A, Sircar R, Heuser C, Tillmann T, Diehl V, Kruis W (1998). Chimeric anti-TAG72 receptors with immunoglobulin constant Fc domains and gamma or zeta signalling chains. Int J Mol Med.

[13] Kim SJ, Hong HJ (2007). Guided selection of human antibody light chains against TAG-72 using a phage display chain shuffling approach. J Microbiol.

[14] Posey AD, Schwab RD, Boesteanu AC, Steentoft C, Mandel U, Engels B (2016). Engineered CAR T cells targeting the cancer-associated tn-glycoform of the membrane Mucin MUC1 control adenocarcinoma. Immunity..

[15] Gutierrez R, Shah PD, Hamid O, Garfall AL, Posey A, Bishop MR (2021). Phase I experience with first in class TnMUC1 targeted chimeric antigen receptor T-cells in patients with advanced TnMUC1 positive solid tumors. J Clin Oncol.

[16] Meril S, Harush O, Reboh Y, Matikhina T, Barliya T, Cohen CJ (2020). Targeting glycosylated antigens on cancer cells using siglec-7/9-based CAR T-cells. Mol Carcinog.

[17] Brown GD, Willment JA, Whitehead L (2018). C-type lectins in immunity and homeostasis. Nat Rev Immunol.

[18] Higashi N, Morikawa A, Fujioka K, Fujita Y, Sano Y, Miyata-Takeuchi M (2002). Human macrophage lectin specific for galactose/N-acetylgalactosamine is a marker for cells at an intermediate stage in their differentiation from monocytes into macrophages. Int Immunol.

[19] Higashi N, Fujioka K, Denda-Nagai K, Hashimoto S, Nagai S, Sato T (2002). The macrophage C-type lectin specific for galactose/N-acetylgalactosamine is an endocytic receptor expressed on monocyte-derived immature dendritic cells. J Biol Chem.

[20] van Vliet SJ, van Liempt E, Saeland E, Aarnoudse CA, Appelmelk B, Irimura T (2005). Carbohydrate profiling reveals a distinctive role for the C-type lectin MGL in the recognition of helminth parasites and tumor antigens by dendritic cells. Int Immunol.

[21] Mortezai N, Behnken HN, Kurze AK, Ludewig P, Buck F, Meyer B (2013). Tumor-associated Neu5Ac-Tn and Neu5Gc-Tn antigens bind to C-type lectin CLEC10A (CD301, MGL). Glycobiology..

[22] Jegouzo SA, Quintero-Martinez A, Ouyang X, dos Santos A, Taylor ME, Drickamer K (2013). Organization of the extracellular portion of the macrophage galactose receptor: a trimeric cluster of simple binding sites for N-acetylgalactosamine. Glycobiology..

[23] Suzuki N, Yamamoto K, Toyoshima S, Osawa T, Irimura T (1996). Molecular cloning and expression of cDNA encoding human macrophage C-type lectin. Its unique carbohydrate binding specificity for Tn antigen. J Immunol.

[24] Nollau P, Wolters-Eisfeld G, Mortezai N, Kurze AK, Klampe B, Debus A (2013). Protein domain histochemistry (PDH): binding of the carbohydrate recognition domain (CRD) of recombinant human glycoreceptor CLEC10A (CD301) to formalin-fixed, paraffin-embedded breast cancer tissues. J Histochem Cytochem.

[25] Hombach A, Wieczarkowiecz A, Marquardt T, Heuser C, Usai L, Pohl C (2001). Tumor-specific T cell activation by recombinant immunoreceptors: CD3 zeta signaling and CD28 costimulation are simultaneously required for efficient IL-2 secretion and can be integrated into one combined CD28/CD3 zeta signaling receptor molecule. J Immunol.

[26] Lichtenfels R, Biddison WE, Schulz H, Vogt AB, Martin R (1994). CARE-LASS (calcein-release-assay), an improved fluorescence-based test system to measure cytotoxic T lymphocyte activity. J Immunol Methods.

[27] Li W, Qiu S, Chen J, Jiang S, Chen W, Jiang J (2020). Chimeric Antigen Receptor Designed to Prevent Ubiquitination and Downregulation Showed Durable Antitumor Efficacy. Immunity..

[28] Arribas J, Coodly L, Vollmer P, Kishimoto TK, Rose-John S, Massague J (1996). Diverse cell surface protein ectodomains are shed by a system sensitive to metalloprotease inhibitors. J Biol Chem.

[29] Moss ML, Rasmussen FH (2007). Fluorescent substrates for the proteinases ADAM17, ADAM10, ADAM8, and ADAM12 useful for high-throughput inhibitor screening. Anal Biochem.

[30] Kenny PA, Bissell MJ (2007). Targeting TACE-dependent EGFR ligand shedding in breast cancer. J Clin Invest.

[31] Marcelo F, Supekar N, Corzana F, van der Horst JC, Vuist IM, Live D (2019). Identification of a secondary binding site in human macrophage galactose-type lectin by microarray studies: Implications for the molecular recognition of its ligands. J Biol Chem.

[32] Fedorov VD, Themeli M, Sadelain M (2013). PD-1- and CTLA-4-based inhibitory chimeric antigen receptors (iCARs) divert off-target immunotherapy responses. Sci Transl Med.

[33] Zhang C, Zhuang Q, Liu J, Liu X (2022). Synthetic biology in chimeric antigen receptor T (CAR T) cell engineering. ACS Synth Biol.

[34] Wagner DL, Fritsche E, Pulsipher MA, Ahmed N, Hamieh M, Hegde M (2021). Immunogenicity of CAR T cells in cancer therapy. Nat Rev Clin Oncol.

