# Supplementary material for: Glyco-binding domain chimeric antigen receptors as a new option for cancer immunotherapy
Source: Gene Ther. 2022 Dec 19;30(7-8):603–11. doi: 10.1038/s41434-022-00374-x (PMC10457195; doi:10.1038/s41434-022-00374-x)
Supplement: Supplementary file 1 — Supplemental material and methods [file 41434_2022_374_MOESM1_ESM.docx]

**Supplementary material and methods**

*Sequences of primers used for CD301-CAR cloning*

CD301-CAR

P1: 5` atcagtgcctcagtcatcatgtcgcgagaagggacctgctgccc 3`

P2: 5` atttgggctcggcgggatccccgtgactctcctggctgg 3`

CD301-linker-CAR

P1: 5` atcagtgcctcagtcatcatgtcgcgagaagggacctgctgccc 3`

P2: 5` atttgggctcggcgggatcccccgaccctccgcctccgctaccgcctccaccagagcctcctcccccgtgactctcctggctgg 3`

CD301-linker-myc-CAR

P1: 5` atcagtgcctcagtcatcatgtcgcgagaacaaaaactcatctcagaagaggatctggaagggacctgctgccc 3`

P2: 5` atttgggctcggcgggatcccccgaccctccgcctccgctaccgcctccaccagagcctcctcccccgtgactctcctggctgg 3`

*Cell lines*

HEK293T cells and the breast cancer cell lines MCF7, T47D, MDA-MB-231, MDA-MB-468, KPL1 and SK-BR3 were cultured in DMEM medium, while RPMI 1640 medium was used for BT474 and K562 Both media (Gibco, Thermo Fisher Scientific). were enriched with 10% heat-inactivated fetal bovine serum (FBS, Gibco, Thermo Fisher Scientific). NK92 cells were grown in alpha MEM medium (Sigma Aldrich) supplemented with 12.5% horse serum and 12.5% FBS, 100 IU/mL hIL-2 (Proteintech). For cultivation of MCF10A cells the MEGM™ Mammary Epithelial Cell Growth Medium BulletKit™ (Lonza) supplemented with 100 ng/ml cholera toxin (Merck) without gentamycin was used. Neuroblastoma cell lines SK-NSH, SK-N-AS and Kelly were cultivated in RPMI with 10% heat-inactivated fetal bovine serum. 2 mM L-Glutamine, 100 U/mL penicillin and 100 μg/mL streptomycin (all from Gibco) were added to all media and the cell lines were maintained at 37°C in a humidified 5% CO2 incubator.

The identities of the cell lines MCF7, T47D, MDA-MB-231, MDA-MB-468, MCF10A, K562, Kelly, SK-N-SH, SK-N-AS were recently confirmed by STR analysis (July and January 2022).

*FACS Analysis of breast cancer cell lines*

Cells were stained at 4°C using FITC-labelled recombinant CD301. The generation of recombinant CD301 was described before (21). For fluorescent labeling cell culture supernatant containing recombinant CD301 was preincubated with biotinylated anti-myc antibody (clone 9E10, Santa Cruz Biotechnology) over night at 4°C. Streptavidin-FITC was added for one hour at RT. Cells were washed with binding buffer (PBS containing 1 mM CaCl_2_ and 2 mM MgCl_2_) and incubated with labeled CD301 for one hour at 4°C. After washing, cells were analyzed in MACS Quant 10 flow cytometer (Miltenyi).

*Glycan binding assay*

For the analysis of binding properties of the CD301-CAR constructs biotinylated polyacrylamide glycoconjugates (Lectinity) were labeled with streptavidin-alexa 647 with a molecular ratio of 1:0.25 for one hour at RT. Aminoglucitol, the negative control, consists of the biotinylated PAA backbone without a glycan structure. Binding of glycoconjugates to 1x10^5^ NK92 CAR or wildtype cells was performed in binding buffer containing 1 mM CaCl_2_ and 2mM MgCl_2_ for one hour at 4°C. After washing, cells were analyzed by flow cytometry.

Degranulation assay

Degranulation of NK92 CAR and wildtype NK92 cells was induced upon interaction with target cells at E:T ratio of 1:1 for 4 h at 37°C, and was assessed by measuring expression of CD107a with an PE/Cy7 -labelled anti-CD107a antibody (Biolegend, #328617) at the surface of FITC labelled anti-CD56 stained NK92 cells (Miltenyi, #130-114-740). Effector cells stimulated with PMA/ionomycin or kept without targets served as controls.

Cytokine Release Assay

Interferon gamma (IFN-γ) production was determined by an ELISA IFN-γ Screening Set (Thermo Fisher Scientific), according to the manufacturer’s instructions. Briefly, 1×10^5^ effector cells were seeded in triplicates together with 1×10^5^ target cells, in 96-well round bottom plates. Cytokine secretion was measured after 16h of incubation using a Varioscan (Thermo Fisher Scientific).


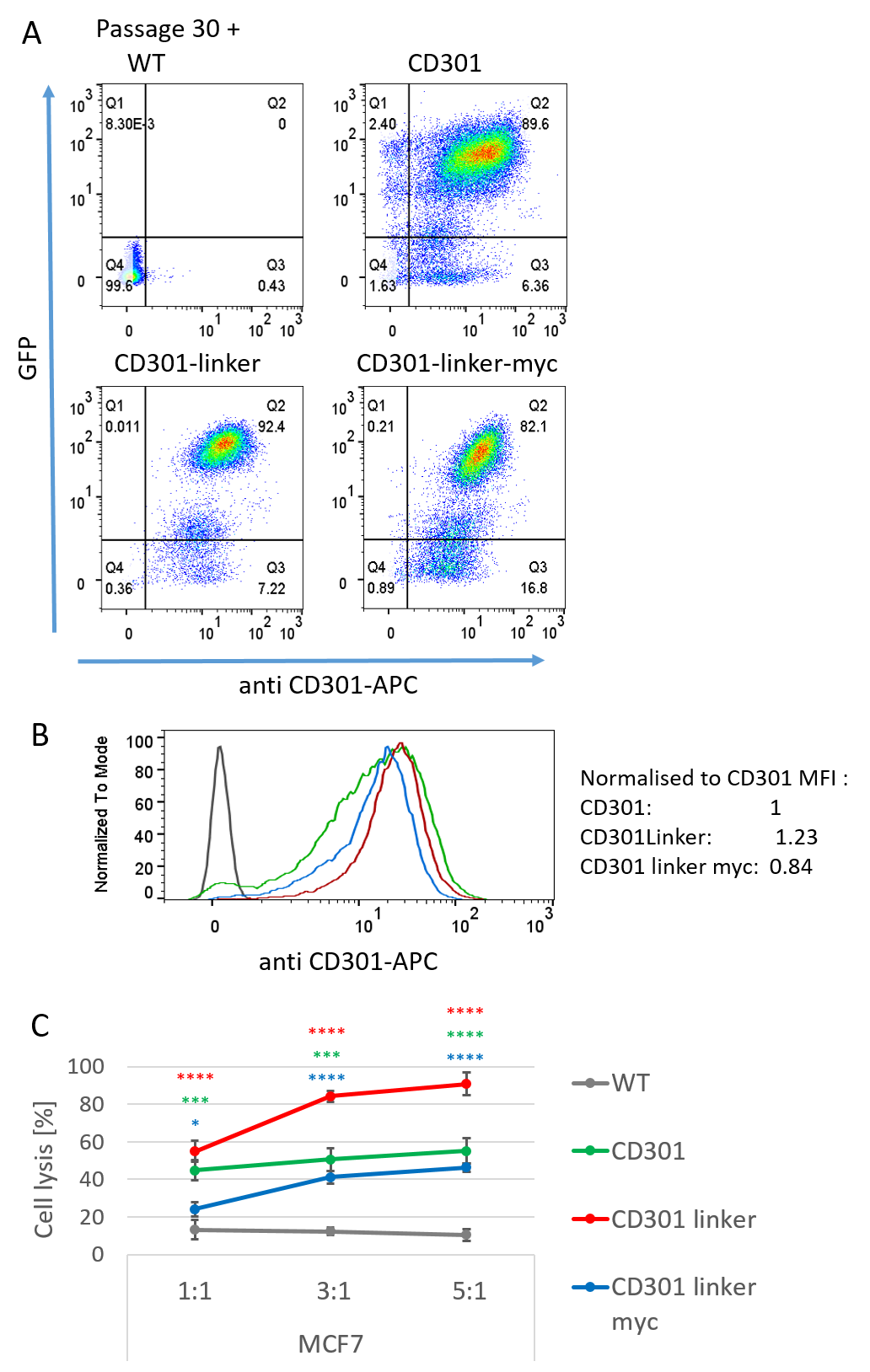


**Supplemental Figure 1: Expression of CD301 CAR constructs NK92 cells with high passage number** A) WT and CAR expressing NK92 cells were analyzed by flow cytometry using an anti-CD301- APC antibody specific for CD301 CRD and GFP reporter expression. B) Overlay histogram of CD301 CAR expressions. CAR expression was analyzed by flow cytometry using an anti-CD301-APC antibody. Expressionlevels normalized to the expression of the CD301 CAR are given.on the right. C) Cytotoxicity measurement of CAR expressing NK92 with high passagae number. Columns represent the median of triplicates Error bars show standard deviation. p < 0.05, p < 0.01 or p < 0.001 p < 0.0001 were indicated by *, **, *** or **** respectively.


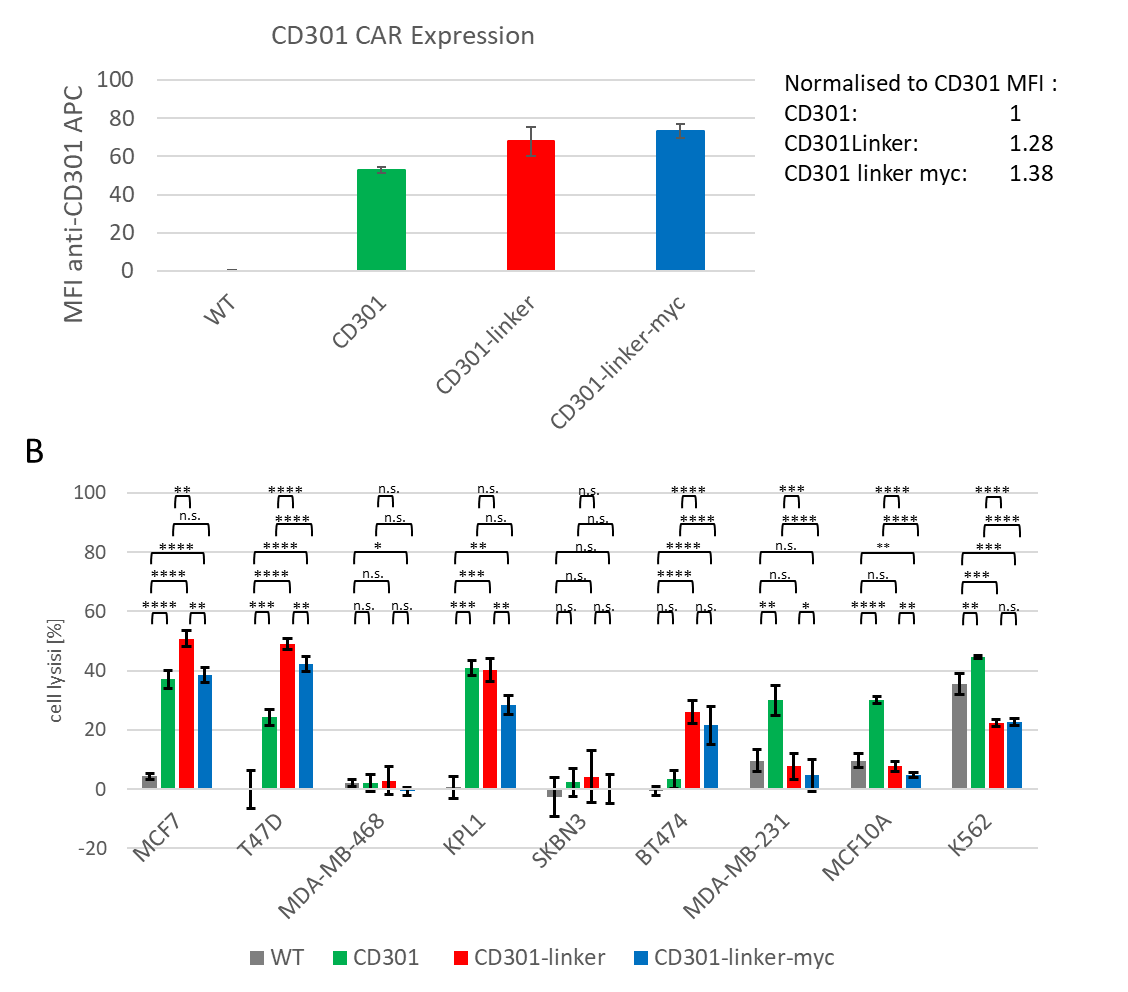


**Supplemental Figure 2: Cytotoxicity normalized to CAR expression level.** A) CAR expression was analyzed by flow cytometry using an anti-CD301-APC antibody in triplicates Expressionlevels normalized to the expression of the CD301 CAR are given.on the right. B) Cytotoxicity measurement normalized to CD301 CAR expression. Columns represent the median of triplicates. Error bars show standard deviation. p < 0.05, p < 0.01 or p < 0.001 p < 0.0001 were indicated by *, **, *** or **** respectively.


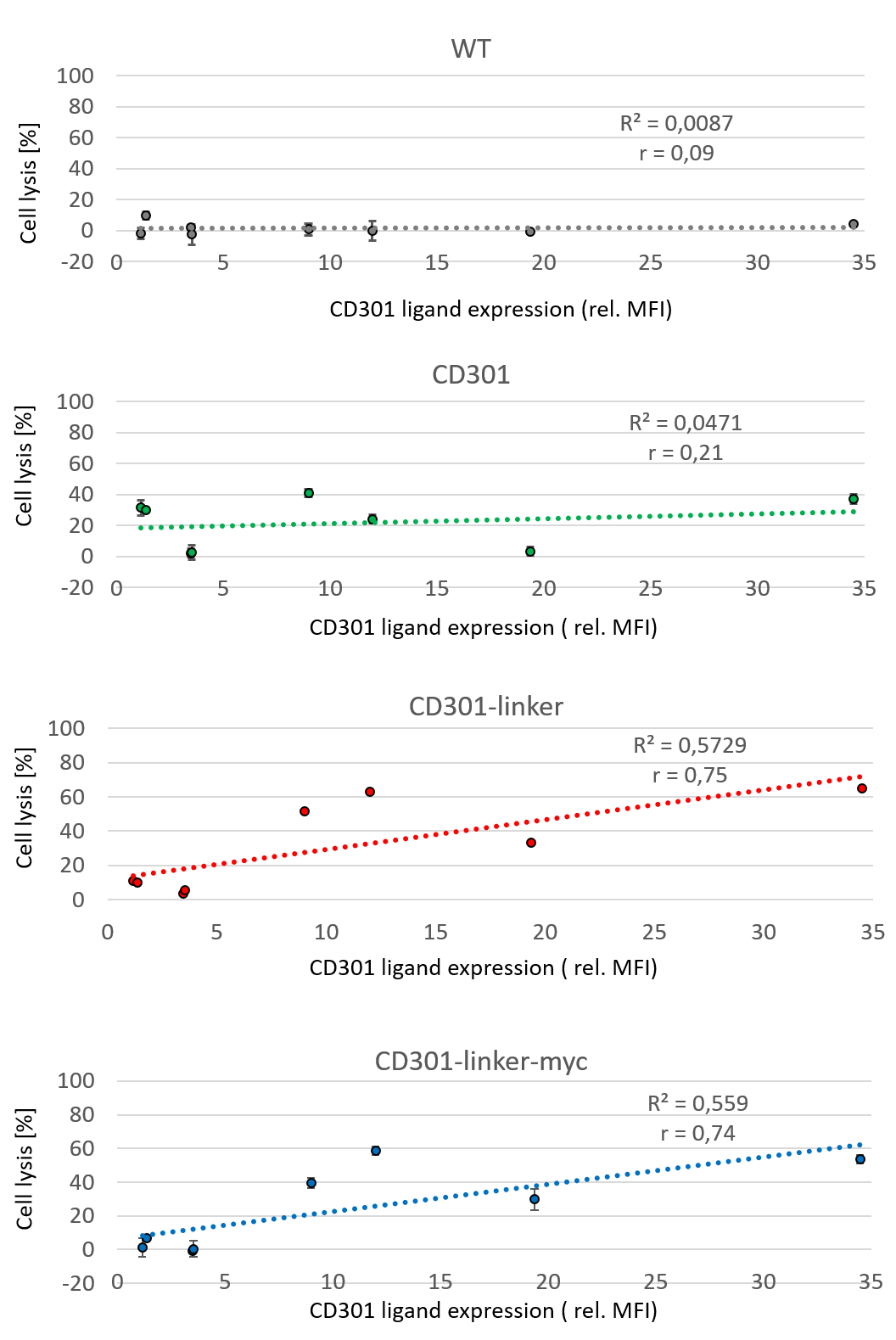


**Supplemental Figure 3: Correlation of CD301 ligand expression of breast cancer cell lines with killing rate of CAR NK92 cells:** Percentages of cell lysis from cytotoxicity assays were depicted in correlation to the expression of CD301 ligands measured by flow cytometry using recombinant CD301. Coefficient of determination (R^2^) and Pearson coefficient (r) are shown in the diagrams.


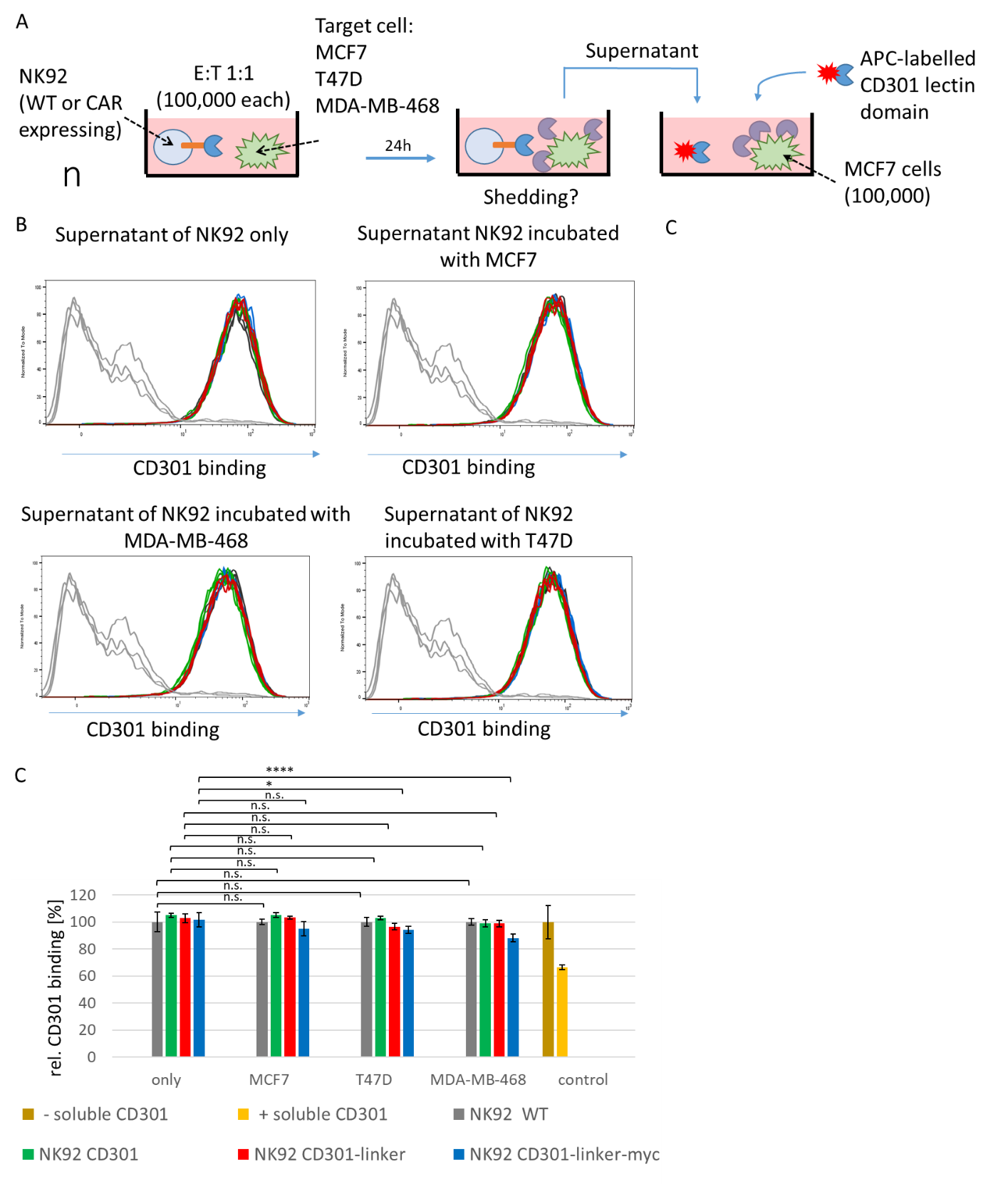


**Supplemental Figure 4: Competitive binding assay with supernatants after 24h of co-culture.** A) Scheme of experimental procedure. NK92 cell (WT or CAR expressing) were incubated with target cells (E:T 1:1) for 24h. Supernatants were added to MCF7 cells before staining with recombinant CD301 occurred. B) Binding of fluorescently labelled CD301 to MCF7 cells was analyzed by flow cytometry. C) For calculation of relative CD301 binding, MFIs of MCF7 were normalized values from MCF7 cells incubated with supernatant from WT NK92 cells. Columns represent the median of triplicates. Error bars show standard deviation. p < 0.05, p < 0.01 or p < 0.001 p < 0.0001 were indicated by *, **, *** or **** respectively.


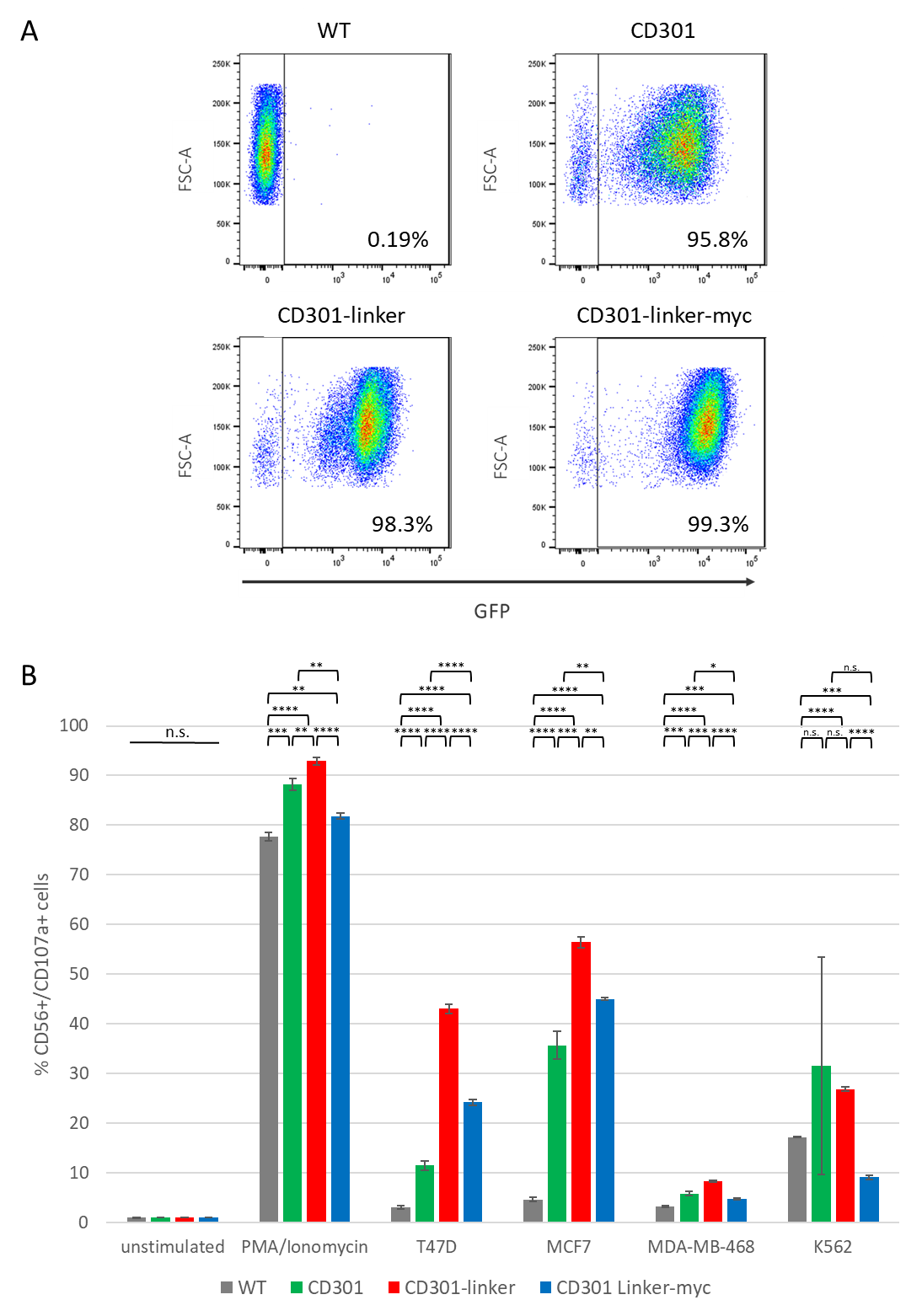


**Supplemental Figure 5: CAR expression leads to enhanced degranulation of NK92 cells upon engagement with CD301 ligand-positive breast cancer cells** A) GFP-positive NK92 cells were measured by flow cytometry and showed comparable frequencies. B) Degranulation of NK92 CAR cells was analyzed by flow cytometry assessment of CD107a surface expression after 4h of co-culture with MCF7, T47D, MDA-MB-468 or K562 cells. Parental NK-92 cells were included for comparison. Unstimulated effector cells or stimulated with PMA/ionomycin served as basal and positive controls, respectively. B) Evaluation of degranulation assay. Columns represent mean percentage of CD107a positive NK92 cells measured in triplicates. Error bars show standard deviation. *p* < 0.05, *p* < 0.01 or *p* < 0.001 p < 0.0001 were indicated by *, **, *** or **** respectively.

**
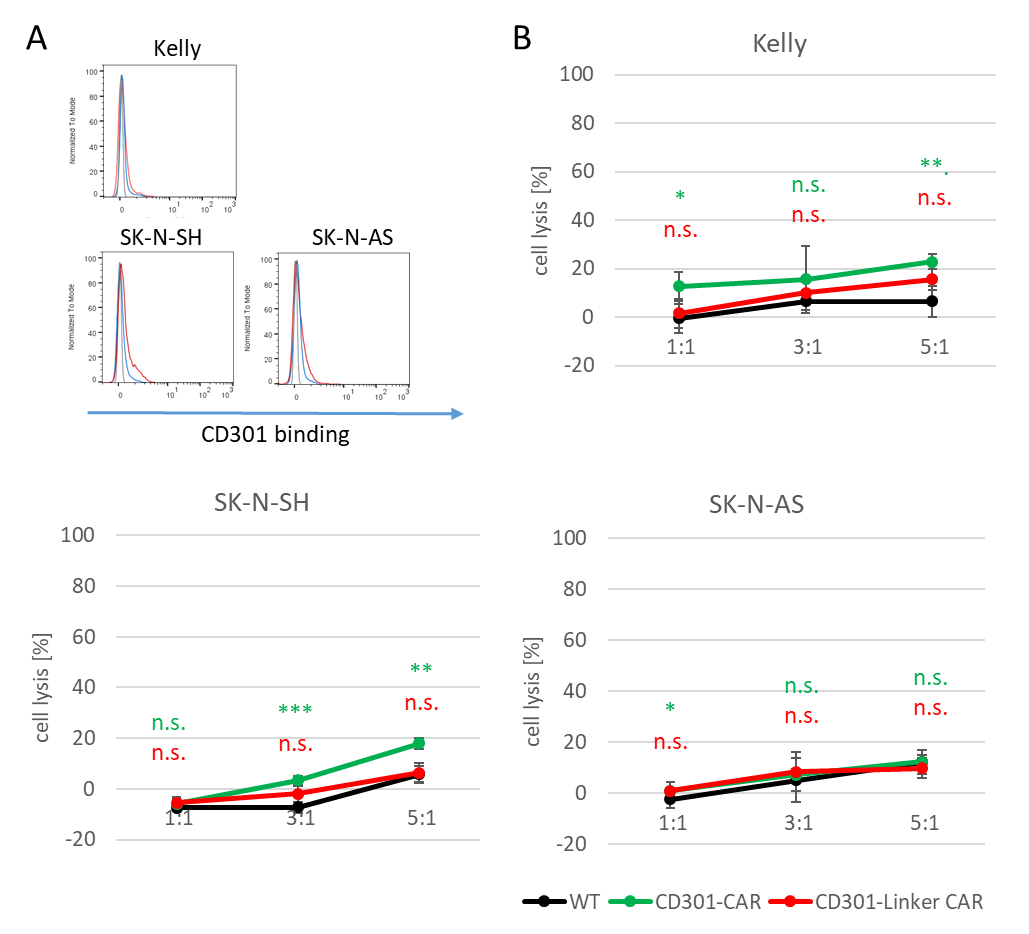
**

**Supplemental Figure 6: Test of CD301-CARs specificities with neuroblastoma cell lines** A) Binding of CD301 conjugates on neuroblastoma cell lines. B) Cytotoxicity measurements. Columns represent mean percentage of cell lysis in % measured in triplicates. Error bars show standard deviation. *p* < 0.05, *p* < 0.01 or *p* < 0.001 p < 0.0001 were indicated by *, **, *** or **** respectively


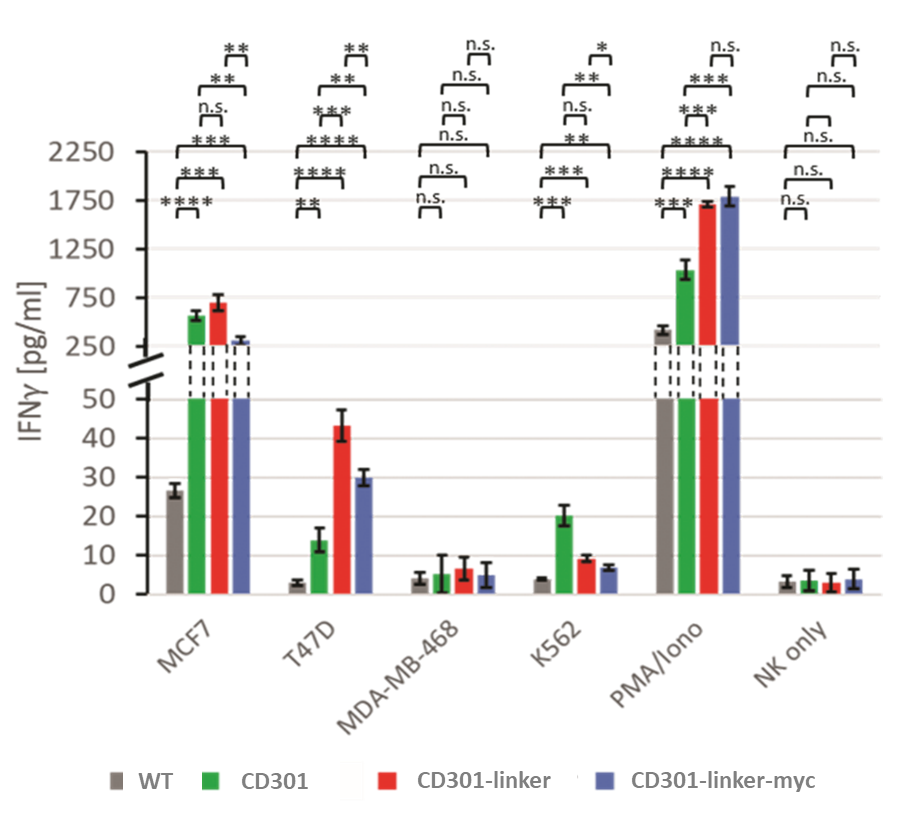


**Supplemental Figure 7: Increased INF-γ secretion of CAR epressing NK92 cells in the presence of ligand-positive target cells.** IFN-γ release was analyzed in supernatants of CAR expressing NK92 CAR or wildtype NK92 cells stimulated with MCF7, T47D, MDA-MB-468 cells (E:T 1:1), respectively. Untreated effector cells and effector cells treated with PMA/ionomycin served as controls. Results are reported as mean values ± SD of triplicates . *p* < 0.05, *p* < 0.01 or *p* < 0.001 p < 0.0001 were indicated by *, **, *** or **** respectively.
